# High Prevalence of Thoracic Aortic Dilatation in Men with Previous Inguinal Hernia Repair

**DOI:** 10.1055/s-0042-1749172

**Published:** 2022-11-01

**Authors:** Emelie Carlestål, Anders Thorell, Lott Bergstrand, Francis Wilamowski, Anders Franco-Cereceda, Christian Olsson

**Affiliations:** 1Department of Molecular Medicine and Surgery, Karolinska Institute, Stockholm, Sweden; 2Department of Cardiothoracic Surgery, Karolinska University Hospital, Karolinska Institute, Stockholm, Sweden; 3Clinical Sciences at Danderyd Hospital, Karolinska Institute, Stockholm, Sweden; 4Department of Surgery, Ersta Hospital, Stockholm, Sweden; 5Department of Radiology, Ersta Hospital, Stockholm, Sweden

**Keywords:** thoracic aortic aneurysm, inguinal hernia, risk factors, screening

## Abstract

**Background**
 Identifying a useful marker for thoracic aortic dilatation (TAD) could help improve informed clinical decisions, enhance diagnosis, and develop TAD screening programs. Inguinal hernia could be such a marker. This study tested the hypothesis that the thoracic aorta is larger and more often dilated in men with previous inguinal hernia repair versus nonhernia controls.

**Methods**
 Four hundred men each with either previous inguinal hernia repair or cholecystectomy (controls) were identified to undergo chest computed tomography to measure the diameter of the thoracic aorta in the aortic root, ascending, isthmic, and descending aorta and to provide self-reported health data. Presence of TAD (root or ascending diameter > 45 mm; isthmic or descending diameter > 35 mm) and thoracic aortic diameters were compared between groups and associations explored using uni- and multivariable statistical methods.

**Results**
 Complete data were obtained from 470/718 (65%) eligible participants. TAD prevalence was significantly higher in the inguinal hernia group: 21 (10%) versus 6 (2.4%),
*p*
 = 0.001 for proximal TAD, 29 (13%) versus 21 (8.3%),
*p*
 = 0.049 for distal TAD, and 50 (23%) versus 27 (11%),
*p*
 < 0.001 for all aortic segments combined. In multivariable analysis, previous inguinal hernia repair was independently associated with dilatation of the proximal aorta (odds ratio 5.3, 95% confidence interval 1.8–15,
*p*
 = 0.003). Contrarily, mean thoracic aortic diameters were similar (root and ascending aorta) or showed clinically irrelevant differences (isthmus and descending aorta).

**Conclusion**
 TAD, but not increased aortic diameters on average, was common and significantly more prevalent in men with previous inguinal hernia repair. Hernia could be a marker condition associated with increased prevalence of TAD. Ultimately, TAD screening could consider hernia as a possible selection criterion.

## Introduction


Thoracic aortic dilatation (TAD) is most often asymptomatic and diagnosed either at acute presentation or incidentally by examinations primarily performed for other reasons. The risk posed by TAD is that of a sudden aortic event; aortic dissection or rupture. Case fatality rate and surgical mortality for acute aortic events are high, approaching 70 and 20%, respectively.
1
2
Surgical mortality in elective TAD operations, on the other hand, is low, 0 to 2%.
3
4
In absence of specific, and relatively uncommon, risk conditions (e.g., Marfan syndrome or family history), surgical indication is often based on aortic diameter. Increased aortic diameter increases the risk of acute events.
5
6
Nevertheless, aortic diameter alone is unreliable and insufficient to accurately predict acute events.
7
Therefore, to diagnose and route more surgical candidates to a strongly preferable elective operation, screening would be valuable. However, TAD prevalence in the normal population is comparatively low, approximately 15/100,000 men
8
and screening would profit from, or even demand, useful selection criteria.



Inguinal hernia is a relatively common condition, with reported lifetime prevalence in men around 27%.
9
Inguinal hernia is much more common in men, related to the embryonal testicular descent through the inguinal canal. Symptomatic inguinal hernia is usually treated by surgical repair. Untreated hernia is associated with risk of ileus and incarceration whereas outcomes of elective surgical repair are excellent.
9
Therefore, previous hernia repair is a useful proxy for inguinal hernia. If associated, inguinal hernia could serve as an easily detected (by clinical examination and history) marker, indicating increased risk of otherwise unsuspected TAD.


This study aimed to explore the association between inguinal hernia and TAD in men. The study hypothesis was that the thoracic aorta is larger in men with previous inguinal hernia repair compared with controls without hernia. This hypothesis was further divided into two specific questions. First, is the prevalence of TAD increased in men with a history of inguinal hernia repair compared with controls? Second, are thoracic aortic diameters increased in men with a history of inguinal hernia repair compared with controls? To test the study hypothesis, diameters of specified segments of the thoracic aorta (root, ascending, isthmus, and descending) and presence of TAD in separate aortic segments and overall were measured by chest computed tomography (CT) and compared between patients with previous inguinal hernia and controls. History of inguinal hernia repair was further assessed by multivariable analysis as an independently associated predictor of TAD.

## Materials and Methods

### Study Population and Study Design

The study was approved by the regional research ethics committee (No 2015/1566–31) and by the local radiation protection committee (No K2402–2015). Written informed consent was obtained from all study subjects.

From January 2016 to September 2018, the four hundred consecutive adult (> 17 years) men with previous (5 years or more) inguinal hernia repair at a single adult surgical unit (Department of Surgery, Ersta Hospital, Stockholm, Sweden) were identified, and those eligible (i.e., alive and not duplicated) subsequently invited to undergo chest CT scan and provide self-reported health data. Similarly, another 400 consecutive men with previous laparoscopic cholecystectomy for symptomatic gallstone disease (cholelithiasis, a condition not known to have any association with connective tissue dysfunction or aneurysm development) at the same unit during the same period of time, were identified as controls. Accordingly, the controls were similar regarding sex, catchment area, timeframe, and presenting type, that is, as elective general surgery patients treated at the same facility. No specific exclusion criteria were applied. The minimum 5-year hiatus between operation and study eligibility was chosen to reproduce the clinical situation of evaluating the risk of TAD in a previously operated (inguinal hernia and cholecystectomy alike) patient and to reflect adequate lead time, that is, time from presence of a marker condition to diagnosis of the target condition (TAD).

Invitations, including study description, health questionnaire, informed consent sheet, and a prepaid response envelope were sent by mail, with follow-up by phone when needed for nonresponders or to expand on study details. The health questionnaire asked the subject to provide numeric data on age, height, and weight and categorical (yes/no if not stated otherwise) data on smoking (present/former/never), hypertension, myocardial infarction or angina, chronic obstructive pulmonary disease, diabetes, connective tissue disease (Marfan, Ehlers-Danlos), abdominal aortic aneurysm (AAA), family history of any aortic aneurysm, and current medications. Study subjects were identified through two existing Swedish nationwide, validated, all-inclusive quality registries: “National Quality Registry for Gallstone Surgery and Endoscopic Retrograde Cholangiopancreatography” and “National Quality Registry for Inguinal Hernia Surgery,” respectively.

### Chest Computed Tomography and Aortic Measurements


**Fig. 1 FI210013-1:**
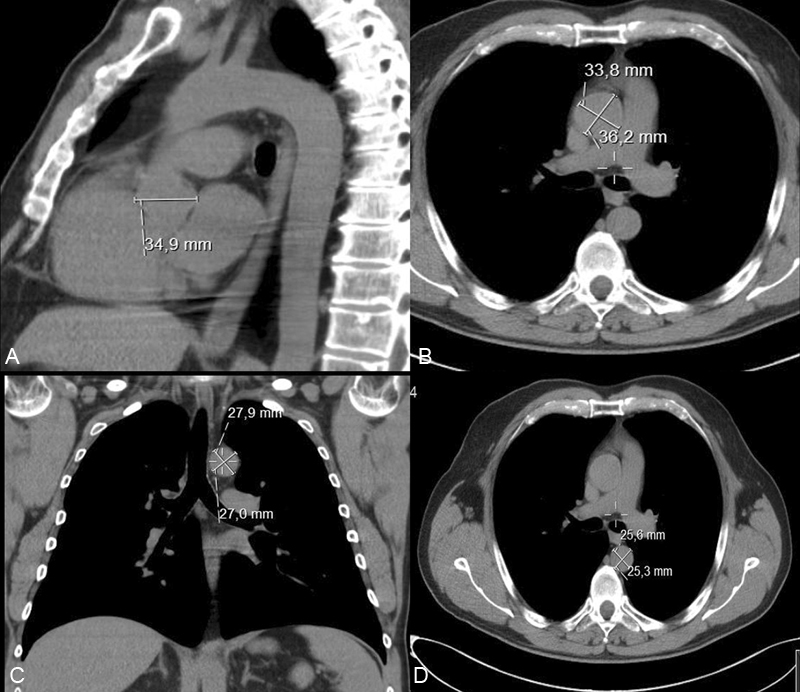
Thoracic aortic diameter measurements in low-dose chest computed tomography without intravenous contrast at (
**A**
) the aortic root (sagittal plane, sinus of Valsalva); (
**B**
) ascending aorta (transversal plane at pulmonary artery bifurcation); (
**C**
) aortic isthmus (coronal plane 10 mm from origin of left subclavian artery); (
**D**
) mid-descending aorta (transversal plane at pulmonary artery bifurcation).


Radiological examinations were performed between October 2016 and April 2019 at a single unit (Department of Radiology, Ersta Hospital, Stockholm, Sweden). They were performed as conventional chest CT with 5 mm axial slices with standard multiplanar (sagittal and coronal) reformations, low dose of radiation (1.6–4.8 mSv), and without intravenous contrast agent. This approach was chosen for study subject safety, but also a part of the study rationale, to reflect a clinical “real-world” scenario comprised of chest CT examinations performed primarily for nonaortic reasons. For the purpose of this study, achieved CT imaging quality (exemplified in
Fig. 1
) was considered adequate and clinically relevant.


Thoracic aortic diameters (in millimeters) were measured in prespecified aortic segments by two independent radiologists blinded to clinical data. The prespecified segments were: the aortic root at the mid-level of the sinuses of Valsalva; the mid-ascending aorta at the level of the pulmonary artery bifurcation; the aortic isthmus just distal to the origin of the left subclavian artery; and the mid-descending aorta at the level of the pulmonary artery bifurcation (
Fig. 1
). The aortic root generated a single measurement from sagittal images, other segments generated two orthogonal measurements. The single largest diameter obtained by any of the two observers in each aortic segment of each patient was selected and compared between the subjects with previous inguinal hernia repair and the control subjects. TAD was defined as aortic diameter > 45 mm for the aortic root and ascending aorta and diameter > 35 mm for the aortic isthmus and descending aorta, respectively. The rationale for using the largest obtained aortic diameter was to avoid undue underestimation of aortic diameter applying a “ruling-in” rather than a “ruling-out” strategy, similar to clinical practice. Cutoff diameters for TAD (> 45 mm for the proximal aorta; > 35 mm for the distal aorta) were chosen to represent an aortic dilatation clearly above upper normal limits
10
11
12
13
and to be of clinical relevance since, in most circumstances, this would entail further radiological study and follow-up, even if criteria for surgical repair are not immediately met. For the proximal aorta, aortic diameter was further indexed to body surface area (calculated using the DuBois and DuBois formula), generating the aortic size index (cm/m
^2^
). The reported
5
threshold for moderate aortic event risk (≥ 2.08 cm/m
^2^
) was applied to define TAD according to the aortic size index.


### Statistical Methods


Numbers were presented as counts with percentages and as means with standard deviations (SDs; for normally distributed variables) or medians with interquartile range (for nonnormally distributed variables). Univariate analyses were performed with Student's
*t*
-test for continuous variables and Pearson's chi-square or Fisher's exact test for categorical variables. Spearman's
*r*
was used to estimate correlation. Multivariable analysis, with TAD as the dependent variable, was performed using logistic regression. Generating a predictive model, variables with
*p*
-value < 0.10 in univariate analysis were included, followed by stepwise backward exclusion to retain significantly (
*p*
 < 0.05) associated variables. Logistic regression results were reported as odds ratios (ORs) with 95% confidence interval. Sample size calculations were performed for prevalence of TAD and for differences in aortic diameters, respectively. For 80% power with
*α *
= 0.05 and assuming 10% TAD in the study group versus 4% in the control group, required sample size was 276 in each group. For 95% power with
*α*
 = 0.01 assuming normal aortic size (34 mm) in the control group and increased size by 1 SD (38 mm) in the study group, required sample size was 38 in each group. Based on power calculations, sample size of 300 in each group was judged desirable, and accommodating for 25% declining study invitation, 400 in each group were identified. All statistical analyses were performed using Stata v16 (Stata Corp., College Station, TX).


## Results


The study participants' characteristics and comorbid conditions, from the self-reporting questionnaire, are presented in
Table 1
.


**Table 1 TB210013-1:** Clinical characteristics of study subjects with previous inguinal hernia repair versus previous cholecystectomy (controls)

	Hernia repair *n* = 216	Cholecystectomy *n* = 254	*p* -Value
Age, years	70 ± 10	66 ± 9	**< 0.001**
Height, cm	179 ± 7	179 ± 7	0.72
Weight, kg	83 ± 11	89 ± 13	**< 0.001**
Hypertension	106 (49)	114 (45)	0.36
Ischemic heart disease	29 (14)	17 (7)	**0.012**
*Smoking* Never Previous Current	99 (46) 107 (50) 9 (4)	130 (52) 109 (43) 13 (5)	0.36
Chronic obstructive pulmonary disease	22 (10)	27 (11)	0.85
Diabetes	25 (12)	32 (13)	0.75
Abdominal aortic aneurysm	5 (2)	1 (0.4)	0.065
Connective tissue disorder	1 (0.5)	1 (0.4)	0.90
Family history of aortic disease	12 (6)	8 (3)	0.19

Note: Mean ± standard deviation (SD) or
*n*
(%).
*p*
-Value using Student's
*t*
-test or Pearson's chi-square test.


**Fig. 2 FI210013-2:**
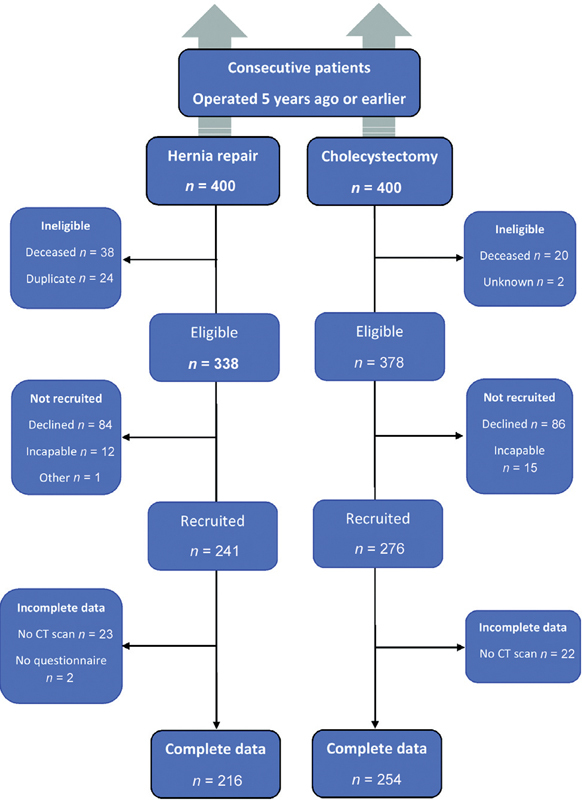
Flowchart with exclusions forming the complete data study groups of subjects with previous hernia repair (
*n*
 = 216) and controls (cholecystectomy,
*n*
 = 254).


Overall, groups were comparable, but subjects in the inguinal hernia repair group were slightly older, weighed less, and more often had self-reported ischemic heart disease compared with the control (cholecystectomy) group. Mean elapsed time from operation to study participation was 8.5 years in the hernia repair group versus 10.5 years in controls. A total of 470/800 (59%) participants finalized the study with complete data: Two hundred sixteen (216/400, 54%) with previous hernia repair and 254/400 (63%) with previous cholecystectomy. However, from study design it followed that 82 men were ineligible: 62 in the hernia group (24 operated twice, 38 dead at the time of study) and 20 in the control group (dead), corresponding to 65% overall participation, 64% (216/338) in the hernia group and 66% (254/380) in the control group (
Fig. 2
).


At the time of identification, 58 men were deceased; 38/58 (65%) operated for inguinal hernia (38/400 = 9.5% of the hernia group) and 20/58 (35%) with cholecystectomy (20/400 = 5% of the control group),
*p*
 = 0.025. Cardiovascular (nonaortic) death was most common in both groups: 15/38 (39%) in the hernia group and 15/20 (75%) in the cholecystectomy group, followed by malignancy (12/38 vs. 4/20). The only aortic death, an infrarenal aneurysm rupture, occurred in the hernia group.



Characteristics of invitees providing questionnaire self-reported health information only, that is, not undergoing CT and thus excluded from analysis of aortic measurements, did not differ significantly from the study participants with complete data, indicating possible random distribution of nonparticipants. Apart from weight (80 ± 9 vs. 92 ± 13 kg,
*p*
 = 0.001) there were no statistically significant intergroup differences (data not shown).


### Thoracic Aortic Diameters


**Fig. 3 FI210013-3:**
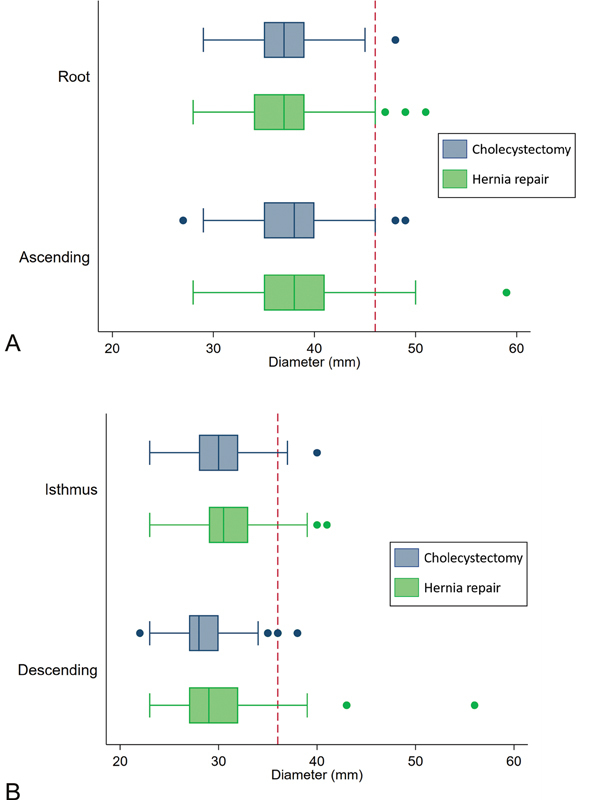
(
**A**
,
**B**
) Boxplot of aortic diameters in millimeters (mm) for aortic segments. Inguinal hernia repair, green; control (cholecystectomy), blue. Thresholds for thoracic aortic dilatation at 46 mm (root and ascending aorta; upper panel) and 36 mm (isthmus and descending aorta; lower panel) indicated by red dashed lines.

As illustrated in
Fig. 3
and detailed in
Table 2
, the hernia repair group had a mean aortic diameter slightly but statistically significantly larger at the isthmus and descending aorta: 31 ± 3 versus 30 ± 3 mm (
*p*
 = 0.008) and 30 ± 4 versus 29 ± 3 mm (
*p*
 = 0.002), respectively. In the proximal aortic segments (root, ascending), there were no statistically significant differences in mean diameters. Overall, interobserver differences were small (1–2 mm differences) and correlation satisfactory (Spearman's
*r*
0.56–0.92), with most divergences and lowest correlation noted for measurements of the aortic root (
Table 3
).


**Table 2 TB210013-2:** Aortic diameters (mm), mean (standard deviation), and range

	Hernia repair *n* = 216	Cholecystectomy *n* = 254	*p* -Value
Aortic root	36.8 (3.7) 28–51	36.5 (3.3) 29–48	0.32
Ascending aorta	38.3 (4.5) 28–59	37.8 (3.9) 27–49	0.24
Aortic isthmus	31.0 (3.1) 23–41	30.2 (2.9) 23–40	0.0075
Descending aorta	29.6 (3.8) 23–56	28.8 (2.8) 22–38	0.015

**Table 3 TB210013-3:** Interobserver differences in aortic diameters per aortic segment

	Median (IQR)	Range	≥ 5 mm difference, *n* (%)	Correlation ( *r* )
Aortic root	2 (1–4)	0–14	91 (19)	0.56
Ascending aorta	1 (1–2)	0–9	11 (2.3)	0.92
Aortic isthmus	2 (1–3)	0–8	27 (5.7)	0.79
Descending aorta	1 (0–2)	0–9	9 (1.9)	0.88

Abbreviation: IQR, interquartile range.

### Thoracic Aortic Dilatations


TAD, on the other hand, was more common in the aortic root with borderline statistical significance (
*p*
 = 0.052) and ascending aorta (
*p*
 < 0.001) of hernia repair subjects (
Table 4
).


**Table 4 TB210013-4:** Prevalence of thoracic aortic dilatation for aortic segments, individual and combined,
*n*
(%)

	Hernia repair *n* = 216	Cholecystectomy *n* = 254	*p* -Value
Aortic root > 45 mm	6 (2.8)	1 (0.4)	0.052
Ascending aorta > 45 mm	14 (6.5)	5 (2.0)	0.013
Proximal aorta	16 (7.4)	6 (2.4)	0.001
Aortic Isthmus > 35 mm	19 (8.8)	15 (5.9)	0.28
Descending aorta > 35 mm	10 (4.6)	6 (2.4)	0.21
Distal aorta	21 (9.7)	19 (7.5)	0.39

Note: Proximal aorta = Root + ascending; Distal aorta = Isthmus + descending.


Dilatation of the proximal aorta, the distal aorta, and all aortic segments combined, respectively, were all significantly more prevalent in the hernia repair group, presenting overall TAD in 50/216 (23%) versus 27/254 (11%),
*p*
 < 0.001. Furthermore, TADs with indication for intervention based on aortic diameter according to current guidelines were encountered in three subjects (3/216, 1.4%), all in the hernia repair group.



Aortic diameter indexed to body surface area (aortic size index) indicated an even more pronounced prevalence of (medium-risk and above) TAD in the hernia group: 31/216 (14%) versus 10/254 (4%),
*p*
 = 0.0001 in the aortic root and 46/216 (21%) versus 32/254 (13%),
*p*
 = 0.012 in the ascending aorta.


### Independent Association between Hernia Repair and TAD


In multivariable analysis, previous hernia repair was found independently associated with ascending aortic dilatation (OR 4.6) and proximal aortic dilatation (OR 5.3), respectively, but not to dilatations of other aortic segments (
Table 5
). Specifically, no variables independently associated with aortic root TAD could be identified: all cases appeared in the hernia group.


**Table 5 TB210013-5:** Multivariable analysis (logistic regression) of independent predictors for thoracic aortic dilatation in each aortic segment (root, ascending, isthmus, descending) and segments combined (proximal, distal)

	Odds ratio	95% Confidence interval	*p* -Value
*Aortic root:*			
No independent predictors found			
*Ascending aorta:*			
Hernia repair	4.6	1.5–14	0.007
Age, y	1.09	1.03–1.15	**0.003**
Weight, kg	1.07	1.03–1.12	**0.001**
*Proximal aorta:*			
Hernia repair	5.3	1.8–15	**0.003**
Age, y	1.09	1.03–1.15	**0.002**
Weight, kg	1.07	1.03–1.11	**0.001**
*Aortic isthmus:*			
Age, y	1.15	1.09–1.21	***p* < 0.0001 **
Weight, kg	1.05	1.02–1.09	0.005
Abdominal aortic aneurysm	8.7	1.4–57	**0.022**
*Descending aorta:*			
Age, y	1.11	1.04–1.18	**0.001**
Weight, kg	1.05	1.002–1.10	0.042
Distal aorta			
Age, y	1.15	1.10–1.21	**< 0.0001**
Weight, kg	1.05	1.02–1.09	**0.001**
Abdominal aortic aneurysm	6.9	1.1–44	0.042

The 460 chest CT examinations yielded 28 (6.1%) pulmonary findings with indication for further study and/or follow-up. Of these, two (0.4%) were found malignant.

## Discussion

This study is the first of its kind, evaluating the association between inguinal hernia and TAD by comparing aortic diameters and prevalence of TAD in subjects with a history of inguinal hernia repair versus controls undergoing chest CT for this specific purpose in a cross-sectional study design suitable to compare prevalence.

Differences in mean aortic diameters were generally very small, but aortic diameters were statistically significantly larger in the distal parts of the aorta (the isthmus and descending aorta) in the hernia repair group. These differences might be of limited clinical relevance by themselves, and should be interpreted with caution, even if sample sizes sufficed to achieve adequate power. Overall, the findings did not support the study hypothesis of a generally increased thoracic aortic diameter in men with previous inguinal hernia repair.


Much more importantly, TAD was significantly more prevalent in the hernia group, in the root as well as the ascending parts and at a 16/216 (7.4%) prevalence, almost three times as common in the proximal aortic segments compared with controls. In the distal parts of the aorta, differences were smaller and not statistically significant. For the ascending and proximal aorta, inguinal hernia repair was the most prominent (OR 4–5) independent predictor in multivariable analysis, further strengthening the association. Furthermore, TAD with diameters fulfilling criteria for intervention were found only in the hernia repair group. These findings do support the study hypothesis and strongly suggest that prevalence of proximal TAD can be expected to be considerable in a population of 70+ years aged men with a history of inguinal hernia repair, with implications for risk profiling, TAD detection, and screening. For perspective, a TAD (> 4.9 cm) was found on only 12/4129 (0.34%) in a population study,
11
indicating the low yield of unselected population-based screening.



The only other variables independently associated with TAD were age and weight (
Table 5
). These findings were consistent with previous observations of larger aortic diameter with increasing age
10
11
12
13
14
and body surface area.
5
11
Accordingly, indexing the aortic diameter to body surface area produced even more pronounced differences, since weight (but not height) was higher in the control group. Self-reported presence of AAA was associated only with isthmic or distal aortic dilatation. This association has been described previously
15
and has not been suggested to encompass the proximal aorta.



Several previous studies have reported an association between inguinal and other abdominal wall hernias including incisional hernias, with AAA,
16
17
but in a large population-based study such an association could not be confirmed.
18
While still interesting from a pathophysiological mechanistic perspective, a relationship between hernia and AAA is of relatively less clinical importance, since full-scale AAA screening programs are already operational in risk groups defined by sex and age. In a cohort (
*n*
 = 1,051) of patients undergoing surgery for TAD
19
a higher-than-expected prevalence of inguinal hernia as well as a larger proportion of inguinal hernia in patients undergoing proximal aortic surgery compared with isolated aortic valve surgery was reported, indicating a possible association. Recently, a large study used propensity scoring to match 16,933 hernia patients to 50,799 controls and found a higher incidence of TAD (6.4 vs. 4.8/10,000 person-years,
*p*
 = 0.03) in the hernia group.
20
Such population-based epidemiological studies are based on administrative data sources and coded diagnostic data not intentionally collected to analyze specific queries, but certainly corroborate findings of smaller, clinical, hypothesis-testing studies.



Molecular mechanistic studies suggest several similarities between connective tissue deficiencies in hernia and aneurysmatic aortic wall, but there is not yet sufficient evidence to support a common molecular mechanistic pathway for hernia and TAD.
21



Presently, hernia or history of hernia repair is not recognized as a risk factor in current TAD guidelines
22
but could be considered in forthcoming revisions. In contrast, hypertension and smoking are also associated with TAD, but prevalent in 67 to 86% and 51 to 62%, respectively,
1
23
and, although important associated conditions, therefore less useful as discriminating features to guide screening. Known risk factors for TAD such as Marfan syndrome and family history, on the other hand, are insufficiently prevalent to help guide selective screening in normal populations, and, when identified, already subject to dedicated follow-up. Simple renal cysts have been identified in 38 to 57% of patients with TAD (compared with 15% in non-TAD) and could also serve as an interesting marker,
24
but unlike hernia, renal cysts cannot be detected by clinical examination and medical history but require abdominal CT or ultrasound for detection.


### Study Limitations

In the present study, only men were included, because of their much higher prevalence of inguinal hernia. Women can suffer from inguinal and other abdominal wall hernias, and future studies evaluating a correlation between hernia and TAD in women would be important to definitively establish inguinal hernia as being associated with TAD in both sexes.


Importantly, aortic valve cuspidity was not known in this study. Bicuspid aortic valves (BAVs) are associated with dilated aortic root and ascending aorta. On the other hand, with BAV, diameters of the distal aorta are normal or even smaller than with a tricuspid aortic valve.
25
In this study, diameters of the distal aorta were significantly larger (
Fig. 2
) and distal TAD more common in the hernia repair group (
Table 3
), suggesting an effect not driven by presence of BAV.


## Conclusion

In conclusion, inguinal hernia, or its proxy previous hernia repair, could serve as a marker to help make informed decisions about screening or diagnostic TAD workup to promote TAD detection and timely repair. Study findings supported the hypothesis of increased prevalence of TAD in men with inguinal hernia. Notwithstanding its clinical relevance, further mechanistic studies are needed to understand the relationship better.

## Editor's Commentary


In their beautifully planned and executed study, Carlestål and colleagues have shown that inguinal hernia may well be a marker for ascending aortic disease. This is an important observation that can help to identify asymptomatic ascending aneurysm patients in the general population. This fits in with other “Guilty Associates” that we have identified, which should prompt suspicion of and investigation for ascending aneurysm disease.
1


The overall findings in the paper from Sweden are so strong that I am not concerned by the lack of a difference in average diameters. The prevalence of aneurysm was simply not high enough to be reflected in mean diameters of the two groups.


The aortic root is difficult to measure and, as we have written, definitions and techniques of assessing diameter in this zone are nonuniform and problematic.
2
I believe the method of estimation used by the authors, and illustrated in
Fig. 1
, is appropriate for this type of study.


We thank the Swedish group for this important report. We are also grateful to the patients for submitting to a limited CT scan just for the purposes of this investigation.

## References

1 Elefteriades JA, Sang A, Kuzmik G, Hornick M. Guilt by association: paradigm for detecting a silent killer (thoracic aortic aneurysm). Open Heart 2015;2(01):e000169

2 Elefteriades JA, Mukherjee SK, Mojibian H. Discrepancies in measurement of the thoracic aorta: JACC review topic of the week. J Am Coll Cardiol 2020;76(02):201–217
